# Faculty development in family medicine education: what is needed?

**DOI:** 10.11604/pamj.2017.26.141.9069

**Published:** 2017-03-14

**Authors:** Brian Johnson, William Edward Cayley, Bich-May Nguyen, Paul Larson, Maria del C Colon-Gonzalez, Christine Gibson, Ann Evensen

**Affiliations:** 1 Contra Costa Family Medicine Residency, University of California-San Francisco Northern California Faculty Development Fellowship, USA; 2 Department of Family Medicine and Community Health, University of Wisconsin School of Medicine and Public Health, USA; 3 Department of Family and Community Medicine, Baylor College of Medicine, USA, Memorial Family Medicine Residency, USA; 4 Department of Family Medicine, University of Pittsburgh, USA; 5 Department of Family and Preventive Medicine, University of Texas Rio Grande Valley, USA; 6 Department of Family Medicine, University of Calgary, USA; 7 Department of Family Medicine and Community Health, University of Wisconsin School of Medicine and Public Health, USA

**Keywords:** Faculty development, family medicine, primary care

## Abstract

A growing number of countries are embracing graduate training in the specialty of Family Medicine as a core component of global health systems reform. One significant challenge for new programs is to adequately prepare for educational excellence and leadership. Promising residents are often encouraged to remain in their program as faculty, but may not have had the benefit of specific training in teaching, curriculum development, learner assessment or educational leadership. Faculty Development is a potential avenue to providing these skills to new Family Medicine Faculty and to encourage new graduates to consider teaching. We are currently seeking to further clarify what the current needs and future possibilities are for Family Medicine Faculty Development in Sub-Saharan Africa.

## Commentary

In 2009 the World Health Assembly resolved that primary care health systems should include family physicians and academic and government leaders in Sub-Saharan Africa have advocated for a larger Family Medicine pipeline [1–3].

New Family Medicine residencies in low-income countries are expanding and often are started with the assistance of faculty from higher-income nations. Several countries have developed residency curricula to train new medical school graduates or re-train practicing physicians as Family Medicine practitioners [4–7]. These residencies aim to prepare locally-trained, competent family physicians who will meet the nearly universal lack of post-graduate trained primary care specialists working in underserved areas of their countries. One significant challenge of these new programs is to find family medicine-trained faculty and to adequately prepare them for educational excellence and leadership [3]. Promising senior residents are encouraged to remain in their programs as faculty. However, they may not have had the benefit of formal training in teaching and curriculum development.

Faculty development has been a central part of Family Medicine since the early years of the specialty. Initially focusing simply on teaching in primary care medicine [8], faculty development in Family Medicine has broadened to include “research, administration and career management” [9] and organisational and leadership development skills [10]. Two reviews have highlighted the wide diversity of faculty development programs that have evolved [8, 11]. These reviews also highlight the need for faculty development efforts to be flexible and adaptable to changing needs, demands and contexts. This flexibility is illustrated well even in countries where Family Medicine is an established discipline and where the depth and breadth of faculty development programs vary considerably [8].

We conducted a literature search for published articles describing faculty development in countries where Family Medicine is an emerging specialty. Several studies have described the international development of Family Medicine. Additionally, there is a small but growing body of literature addressing needs for faculty development. A survey at a medical school in Singapore found clinical faculty felt a need for further knowledge in lecture, small group, and clinical teaching, and in teaching house officers and medical officers. Objective structured clinical examination (OSCE) and assessment of professional behavior were two items in which participants wanted much higher knowledge [12]. A faculty needs assessment conducted in India indicated interest in the areas of curriculum design, instructional delivery, student assessment and educational management [13]. A survey of medical educators in China found significant interest in research, management, and especially medical education, with particular interest in learning via educational collaborations or international study [14]. Published descriptions of faculty development interventions include: a workshop series in Nepal focusing on teaching-learning methods, media, microteaching and evaluation techniques [15], a training workshop on effective teaching methods, feedback, knowledge assessment, and time management conducted in Iran [16], a series of faculty development workshops conducted in Saudi Arabia [17], and an overseas training program to specifically support family medicine faculty development for educators from Egypt [18].

Nonetheless, few studies have described clear faculty development competencies and curricula to maintain and support Family Medicine educators. There is a particular lack of information about faculty development in Sub-Saharan Africa. De Villiers and Hellenberg have described the evolution of family medicine in South Africa, including the challenges of establishing the role and value of the discipline in a low-resource country and the reorientation of Family Medicine teachers, trained in a biomedical paradigm, to a patient-centered approach [19, 20] Furthermore, Family Medicine in Nigeria is described as encompassing family care dynamics, primary medical care, and facility-based care either in clinics or in hospitals [21]. While these papers provide descriptors of Family Medicine and the challenges to developing a clear Family Medicine identity in Africa, they do not identify faculty development competencies.

It has been argued that the African situation is distinct enough from the “industrialized world” that the commonly articulated characteristics of Family Medicine may not provide the appropriate training model for African contexts [22, 23]. Efforts to develop a uniquely African approach to family medicine have been growing in recent years [24–26]. We acknowledge that the sub-saharan African region includes many countries with diverse cultures and needs, and one solution is not likely to be sufficient to meet the needs in diverse contexts. Efforts so far have focused on workforce development and trainee education and there is no published literature specifically assessing faculty development needs of African Family Medicine educators.

The scarcity of information on faculty development in areas where Family Medicine is a developing specialty points to the need for further research to both clarify specific, locally relevant faculty development priorities. We will soon report on results of a completed qualitative needs assessment of current faculty in family medicine from countries in sub-Saharan Africa. Once local priorities have been identified it may inform the development of new locally appropriate faculty development programs. Understanding what current and future Family Medicine faculty need to improve their teaching skills may be instrumental to maintaining morale, promoting the specialty, and strengthening the primary care workforce in these countries.
